# Combinatorial Therapy with Long-Acting Tenofovir and Tizoxanide Controls Viral Replication and Liver Inflammation in a Murine AAV-HBV Model of Chronic Hepatitis B

**DOI:** 10.3390/biology15161391

**Published:** 2026-08-14

**Authors:** Mojisola O. Ogunnaike, Ashrafi Sultana, Samiksha Raut, Weimin Wang, Grace Bybee, Howard E. Gendelman, Benson J. Edagwa, Natalia A. Osna, Larisa Y. Poluektova

**Affiliations:** 1 Department of Pharmacology and Experimental Neuroscience, University of Nebraska Medical Center, Omaha, NE 68198, USAhegendel@unmc.edu (H.E.G.); 2 Department of Pathology and Microbiology, University of Nebraska Medical Center, Omaha, NE 68198, USA

**Keywords:** tizoxanide, anti-inflammatory, combination therapy, tenofovir, HBV, nucleoside analogs

## Abstract

Chronic hepatitis B (CHB) infection is a major risk factor for progressive cirrhosis and hepatocellular carcinoma. Persistent virus-induced inflammation alters liver function, leading to accelerated disease and increased mortality. Although lifelong treatment with nucleos(t)ide analogs (NAs) is highly effective at suppressing viral replication, covalently closed circular DNA (cccDNA) persists in hepatocytes to sustain chronic infection, underscoring the need for better interventions and combination therapies. The durable suppression of viral replication and restoration of immune responses through long-acting (LA) therapies offer a promising strategy for sustained HBV control. We transformed tizoxanide (TIZ), a broad-spectrum anti-infective and immunomodulatory agent, into a LA lipophilic prodrug formulation (NM2TIZ) for intramuscular or subcutaneous administration. NM2TIZ exhibited long-term stability during storage and was evaluated in AAV-HBV mice using monotherapy and combination therapy approaches with an LA tenofovir prodrug formulation (NM5TFV). NM5TFV reduced the HBV DNA levels by >2 log_10_ fold. Furthermore, coadministration of NM5TFV with M2TIZ reduced the expression of liver inflammasomes and profibrotic markers. The NM5TFV and NM2TIZ combination reduced HBV replication, inflammation, and fibrogenesis in AAV-HBV-transduced mice.

## Simple Summary

Hepatitis B viral infection (HBV) is a global public health problem threatened by surging infections due to vaccination access, antimicrobial resistance (AMR), climate change, and healthcare worker shortages. Estimates from global health suggest that HBV infection accounts for 300 million chronic infections and 1 million deaths each year, mostly from HBV-related cirrhosis and hepatocellular carcinoma. Existing therapies do not eradicate the virus, and lifelong treatment is required. Since long-acting (LA) therapies have demonstrated improved treatment outcomes in adjacent chronic disease areas, we developed LA prodrug formulations of tenofovir (NM5TFV) and tizoxanide (NM2TIZ) and evaluated their efficacy in immunocompetent mice infected with an adeno-associated viral construct. A single-dose NM5TFV suppressed HBV replication by >2.0 log_10_ for over ten weeks when administered as monotherapy or in combination with monthly doses of NM2TIZ. Co-administration of NM5TFV with NM2TIZ induced anti-inflammatory and anti-fibrotic responses in liver tissues. These findings support the further development of NM5TFV and NM2TIZ combination therapy as a potential treatment for HBV infection.

## 1. Introduction

According to the World Health Organization, over 257 million people worldwide are infected with hepatitis B virus (HBV) [1]. HBV infection remains an emerging healthcare crisis, with more than 887,000 deaths reported annually. Chronic hepatitis B (CHB) remains a significant healthcare challenge in the United States and is most prevalent in Asian and Pacific Islander descendants and African immigrants from sub-Saharan Africa [2]. The highest rates of HBV infection are among persons infected with human immunodeficiency virus (HIV) who inject drugs and men who have sex with men (MSM) [3]. In patients with untreated CHB, the five-year incidence of progression to cirrhosis is 8–20%, with an annual risk of developing hepatocellular carcinoma of 43–62% [4]. Although not curative, TFV-based therapies are considered backbone therapies because of their high potency in achieving virologic control and normalizing liver enzymes; however, burdensome daily oral regimens and inefficient resolution of long-term liver injury remain major challenges for nucleos(t)ide analog (NA) therapies [5]. Tenofovir alafenamide (TAF; Vemlidy^®^), the preferred NA for use against HBV infection, is available as a daily oral therapy and prone to patient noncompliance due to treatment fatigue and other challenges associated with daily oral therapies for chronic illnesses [6]. We have recently developed LA TFV derivatives to improve treatment outcomes through infrequent dosing and durable viral load suppression [7,8]. Notably, NM5TFV prodrug nanosuspension afforded sustained suppression of peripheral HBV DNA (>3 log) following a single intramuscular administration in HBV-transgenic mice [7]. However, the inability of TFV monotherapy to eliminate persistent HBV replication templates (cccDNA), HBsAg antigen burden, immune dysfunction, and liver injury remains a major barrier to achieving a functional cure [9,10]. To address this critical gap, therapeutic efforts have shifted towards multi-mechanistic approaches capable of integrating host-directed, immunomodulatory, or other antivirals along with NA backbone therapy [5,11]. Pharmacological agents with substantial immune-restorative profiles and amenable to LA approaches are promising partners for the proposed combinatorial approaches. In this study, tizoxanide, a broad-spectrum innate immune stimulant, was selected as a complementary partner agent for the LA NM5TFV formulation to improve treatment outcomes.

Initially approved as an antiparasitic immune modulator [12,13], nitazoxanide (NTZ) has been developed to control innate immune responses. NTZ amplifies the host’s innate immune response. It upregulates intracellular antiviral pathways (such as RIG-I and MAVS) and counteracts the strategies used by HBV to evade immune detection. Preclinical studies in HBV-relevant cell models have shown that the active metabolite of NTZ, tizoxanide (TIZ), exerts antiviral effects by interfering with host-dependent processes required for HBV replication, resulting in reductions in HBV DNA and HBsAg, which are the key drivers of HBV persistence [14,15]. Efforts to understand the mechanism of NTZ’s antiviral activity have uncovered a rather broad modulation of multiple cellular pathways, including priming of viral RNA and DNA sensors to amplify cytokine signaling and viral clearance [14,16,17], viral protein glycosylation and transport arrest [18], and rescue of Smc5/6, a host antiviral protein, from Hbx-DDB1-mediated degradation [15,19]. This broad pharmacological activity makes NTZ therapeutically advantageous for the treatment of complex chronic diseases, such as CHB. A few early clinical studies have explored the efficacy of NTZ therapy in seronegative patients as proof of concept for antiviral activity against HBV; however, the short systemic half-life of NTZ and its metabolite, TIZ, necessitated repeated twice-daily oral administration over extended treatment periods (12–24 weeks) to maintain therapeutic drug exposure, highlighting the need for LA therapeutics [17,20,21].

Building on these previously established immunomodulatory properties, we first transformed TIZ into a lipophilic prodrug nanosuspension (NM2TIZ) to improve the apparent half-life of the parent drug and simplify dosing. Thereafter, we investigated whether NM2TIZ provided complementary immune-remediating benefits when co-administered with our previously reported LA NM5TFV formulation in an adeno-associated virus (AAV)-HBV mouse model. The AAV-HBV model can be used to evaluate hepatocyte viral activity in immunocompetent mice [22,23].

## 2. Materials and Methods

### 2.1. Synthesis and Characterization of Prodrugs

#### 2.1.1. Synthesis and Characterization of M2TIZ

M2TIZ was synthesized via a single-step esterification reaction of TIZ with stearoyl chloride (Sigma Aldrich, St. Louis, MO, USA). Specifically, one equivalent of TIZ was dissolved in anhydrous dimethylformamide (DMF) (Sigma-Aldrich Inc., St. Louis, MO, USA) and cooled to 4 °C with continuous stirring. Subsequently, two equivalents of N, N-diisopropylethylamine (DIEA)(Sigma-Aldrich Inc., St. Louis, MO, USA) were added dropwise to the reaction flask. In a separate vessel, stearoyl chloride was dissolved in DMF (5 mL) and added slowly to the cooled reaction mixture. The reaction mixture was then heated to 45 °C and stirred until completion, as monitored by thin-layer chromatography (TLC). Upon completion of the reaction, the solvent was removed under reduced pressure using a rotary evaporator (BÜCHI Labortechnik AG, Flawil, Switzerland). The crude product was purified using silica gel column chromatography(Sorbent Technologies, Norcross, GA, USA). The column was packed and eluted with hexane/ethyl acetate (2:1, *v*/*v*) (Thermo Fisher Inc., Waltham, MA, USA), and fractions were monitored by TLC using hexane/ethyl acetate (1:1, *v*/*v*) as the mobile phase. The fractions containing the desired product were combined and concentrated using a rotary evaporator. The purified product was dried under reduced pressure, precipitated from hexane, and further dried under vacuum, producing average chemical yields of 70–75%. All chemical reactions were conducted under an ambient atmosphere, unless otherwise specified. Purified M2TIZ was used for formulation studies. M2TIZ was prepared as an aqueous nanosuspension stabilized with non-ionic surfactants. Specifically, M2TIZ powder was dispersed in endotoxin-free water (pH 6.9) containing Tween 20 (Sigma Aldrich, St. Louis, MO, USA) and polyethylene glycol-3350 (PEG-3350) (Sigma Aldrich, St. Louis, MO, USA) at a weight ratio of 7:1:1 (*w*/*w*/*w*). The resulting microsuspension was processed by high-pressure homogenization using an EmulsiFlex-C3 homogenizer (Avestin Inc., Ottawa, ON, Canada) at 20,000 ± 1000 psi until the desired nanoparticle size was obtained. The resulting nanosuspension (NM2TIZ) was characterized based on particle size, polydispersity index (PDI), appearance, and drug concentration. The hydrodynamic particle size and PDI were determined by dynamic light scattering (DLS) using a Zetasizer Nano-ZS instrument (Malvern Instruments, Worcestershire, UK). The optimized NM2TIZ formulation exhibited a mean particle size of approximately 221 nm, a PDI of 0.30, and a final drug concentration of approximately 190 mg/mL. The formulation stability was evaluated during storage at room temperature by monitoring the changes in particle size, PDI, and drug concentration over time. The chemical stability of the formulation was assessed by monitoring the M2TIZ concentrations during storage. The formulation was readily syringeable during the study. The M2TIZ concentration in the formulation was quantified using reverse-phase UPLC with ultraviolet (UV) detection. Chromatographic separation was performed using a BEH Shield RP18 column (1.7 µm, 2.1 × 100 mm: Waters Corporation, Milford, MA, USA). The mobile phase consisted of 2% aqueous and 98% organic phases under isocratic elution. The flow rate was maintained at 0.25 mL/min, and M2TIZ was detected at a 371 nm wavelength. Under these chromatographic conditions, M2TIZ was eluted at a retention time of approximately 3.6 min. The column temperature was maintained at 45 °C. For sample preparation, aliquots of the NM2TIZ nanosuspension were diluted in 50:50 methanol–DMSO and sonicated to extract M2TIZ from the formulation. The samples were centrifuged, and the resulting supernatant was transferred to ultra-performance liquid chromatography (UPLC) vials for analysis. M2TIZ concentrations were quantified using a calibration curve prepared using known M2TIZ standards.

#### 2.1.2. Synthesis and Characterization of M5TFV

M5TFV was synthesized and characterized as described previously [15]. Briefly, the prodrug derivatization was achieved by esterifying TFV monophenol with octadecanol. The chemical composition of the resulting compound was confirmed by nuclear magnetic resonance (H1NMR, 13C NMR, and 31P NMR). M5TFV was then formulated as an aqueous nanosuspension with polyethylene glycol (PEG-3350) and polysorbate 20 (Sigma-Aldrich Inc., St. Louis, MO, USA) as stabilizing surfactants. The formulation was produced by milling the prodrug suspension using an Avestin EmulsiFlex-C3 high-pressure homogenizer (Ottawa, ON, Canada) to obtain a syringeable aqueous NM5TFV nanosuspension.

### 2.2. Antiviral and Anti-Inflammatory Assessments

Primary human hepatocytes (PHHs) were sourced from the Liver Tissue Cell Distribution System (Minneapolis, MN; Pittsburgh, PA; NIH Contract #HSN276201200017C) and cultured as described previously [24]. Briefly, PHHs were seeded on collagen-coated plates and maintained in the maintenance medium. Cells were infected with HBV (10^7^ genomes/mL) for 24 h and exposed to varying concentrations (0.1, 1.0, 10, 25, 50, and 100 µM) of M2TIZ or NTZ for 48 h; control cells were left untreated. Cellular HBV DNA, HBV RNA, cccDNA, and secreted HBsAg were analyzed using qPCR and ELISA. For anti-inflammatory studies, human monocyte-derived macrophages (MDMs) were pretreated with or without M2TIZ (10 µM) for 4 h, washed, and exposed to HBV-conditioned media. Cells were harvested, and the gene expression profiles of interleukin-1 beta (IL-1β) and tumor necrosis factor alpha (TNF-α) were evaluated using qPCR.

### 2.3. Establishment of AAV-HBV Model and Treatment Scheme

#### 2.3.1. AAV-HBV Model

To establish a suitable HBV-replicating animal model, immunocompetent mice were transduced with an adeno-associated viral vector containing the HBV-D/ayw (serotype 8) genome (Creative Biogene, New York, NY, USA). Briefly, 6–8-week-old C57BL6/J male mice were injected with 1011 vg/mouse of AAV-HBV viral particles. At 2 months post-transduction, the animals were bled, and serum HBV DNA was measured using the COBAS^®^ TaqMan HBV Test (Roche Diagnostics, Rotkreuz, Switzerland), with a lower limit of detection of 340 IU/mL. The treatment drugs were administered and monitored for 14 weeks, after which the animals were euthanized for downstream analyses.

This study was approved by the Institutional Animal Care and Use Committee of the University of Nebraska Medical Center (protocols #18-087-10FC and #23-057-11FC). The animals were housed under pathogen-free conditions and were fed a standard diet.

#### 2.3.2. Treatments

AAV-HBV mice were evenly distributed into the NM2TIZ, NM5TFV, and NM2TIZ + NM5TFV groups, each with five replicates. NM5TFV was administered as a single 400 mg/kg TFV-equivalent dose, whereas NM2TIZ was administered as three monthly doses of 200 mg/kg TIZ-equivalent. The control group did not receive any treatments. Blood samples were collected from the experimental animals over 14 weeks, after which the animals were sacrificed, and liver tissues were collected for tissue analysis. Murine plasma samples were diluted 17.5-fold and subjected to HBV DNA quantification using the COBAS TaqMan HBV Test (Roche Diagnostics, Pleasanton, CA, USA). The detection limit was 350 IU/mL.

### 2.4. Evaluations

#### 2.4.1. Immunohistochemistry

The tissues were fixed with 4% paraformaldehyde (Thermo Scientific, Waltham, MA, USA) overnight at 4 °C and embedded in paraffin (Leica Biosystems, Richmond, IL, USA). Five-micrometer sections were cut from paraffin blocks, mounted on glass slides, and subjected to immunohistochemical staining, as described previously [15]. Rabbit monoclonal antibodies against HBsAg (HBsAg 7556R) were obtained from Novus Biologicals (Centennial, CO, USA), and rabbit polyclonal antibodies against HBcAg (MBS373019) were obtained from Mybiosource (San Diego, CA, USA). Polymer-based horseradish peroxidase–conjugated goat anti-rabbit systems (Abcam, Cambridge, UK) were used as secondary detection reagents and developed with 3,3′-diaminobenzidine (Sigma-Aldrich, St. Louis, MO, USA). All paraffin-embedded sections were counterstained with Harris Hematoxylin (Leica Biosystems, Deer Park, IL, USA). Collagen deposit was visualized using Picro Sirius Red stain (Abcam plc, Cambridge, UK). Bright-field images were obtained using an Echo confocal microscope (BICO company, San Diego, CA, USA) at an original magnification of ×100 and ×200.

#### 2.4.2. HBV DNA and cccDNA Quantification

HBV DNA and cccDNA copy numbers were quantified by RT-PCR as described previously [15]. The DNeasy Blood and Tissue kit from Qiagene (Hilden, Germany) was used to purify total DNA from cells and frozen liver tissue. HBV DNA (2.5 ng) and cccDNA (200 ng) were selectively amplified using the following assay-specific primer-probes from IDT: HBV DNA, Forward CACCTCTCTTTACGCCCACT, Reverse CGACGTGCAGAGGTGAAG Probe FAM/ATCTGCCCGG/ZEN/ACCGTGCAC/3IABkFQ; HBV cccDNA Forward CTTCTCATCTGCCGGACC Reverse CACAGCTTGGAGGCTTGA Probe FAM/AGGCTGTAG/ZEN/GCATAAATTGGTCT/3IABKfQ. All amplifications were performed using the StepOnePlus RT-PCR System (Applied Biosystems, Foster City, CA, USA). Absolute copies were extrapolated from the serially diluted HBV monomer plasmids.

#### 2.4.3. Quantitative PCR Assays

Total RNA was isolated from the cells and liver tissues using Trizol Reagent (Thermo Fisher Scientific, Asheville, NC, USA). A 2-step procedure was applied, in which 400 ng RNA was reverse-transcribed to cDNA using a high-capacity reverse transcription kit. The cDNA was then amplified using TaqMan Universal Master Mix-II with fluorescent-labeled primers (Applied Biosystems, Foster City, CA, USA). After incubation in a Model 7500 RT-qPCR thermal cycler (Applied Bioscience, Foster City, CA, USA), the relative quantity of each RNA transcript was calculated using its threshold cycle (Ct) after subtracting that of the reference cDNA (*GAPDH*). The data are expressed as the relative quantity of transcripts. TaqMan probes and qPCR primers for transforming growth factor-beta (TGF-β), interleukin-18 (IL-18), IL-1β, TNF-α, and NLR family pyrin domain-containing 3 (NLRP3) were used.

### 2.5. Statistical Analysis

Data analysis was performed using the GraphPad Prism 10.5.0 statistical package. Differences in means were analyzed using an unpaired *t*-test, one-way analysis of variance (ANOVA), and two-way ANOVA. Statistical significance was set at *p* < 0.05.

## 3. Results

### 3.1. M2TIZ Synthesis and Stability Were Evaluated

**Figure 1:**
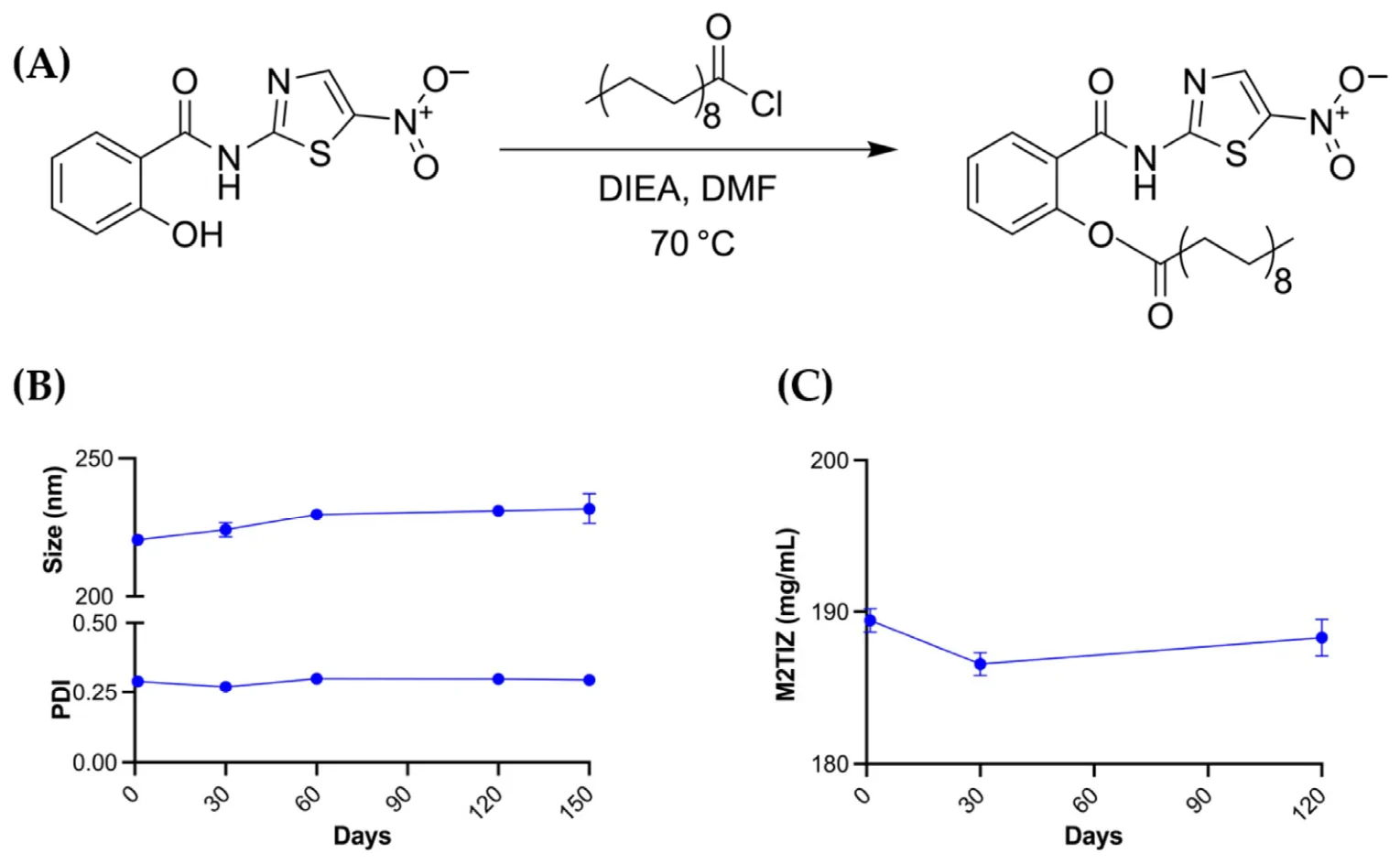
Prodrug synthesis and characterization of NM2TIZ nanosuspension: (**A**) Synthetic scheme illustrating the preparation of the lipophilic tizoxanide prodrug, M2TIZ, via esterification of the parent drug with stearoyl chloride. (**B**) Stability of NM2TIZ nanosuspensions during storage. The particle size distribution and polydispersity index (PDI) were evaluated over time using dynamic light scattering (DLS). (**C**) M2TIZ concentration in the nanosuspension during storage, as determined by UPLC. Data are expressed as mean ± SEM (*n* = 3). The formulation maintained its physicochemical stability throughout the study.

To transform TIZ into an LA injectable depot formulation, a lipophilic prodrug, M2TIZ, was synthesized by esterifying the parent drug with stearoyl chloride (Figure 1A). The purified prodrug was subsequently formulated as an aqueous NM2TIZ nanosuspension and evaluated for its physicochemical stability. The stability of the NM2TIZ formulation was assessed by monitoring the particle size and polydispersity index (PDI) during storage (Figure 1B). Throughout the study period, the nanosuspension maintained a consistent particle size distribution with a mean hydrodynamic diameter of approximately 221 nm and a PDI of 0.30, indicating the preservation of colloidal stability and formulation homogeneity of the nanosuspension. The chemical stability of the formulation was determined by quantifying the M2TIZ concentrations over time (Figure 1C). The proof-of-concept formulation was produced at a drug concentration of 190 mg/mL and remained stable throughout the storage period, demonstrating prodrug stability within the formulation. Collectively, these findings demonstrate the compatibility of the M2TIZ prodrug with scalable, aqueous-based formulation approaches.

### 3.2. NM2TIZ Exhibits Antiviral and Anti-Inflammatory Activities

**Figure 2:**
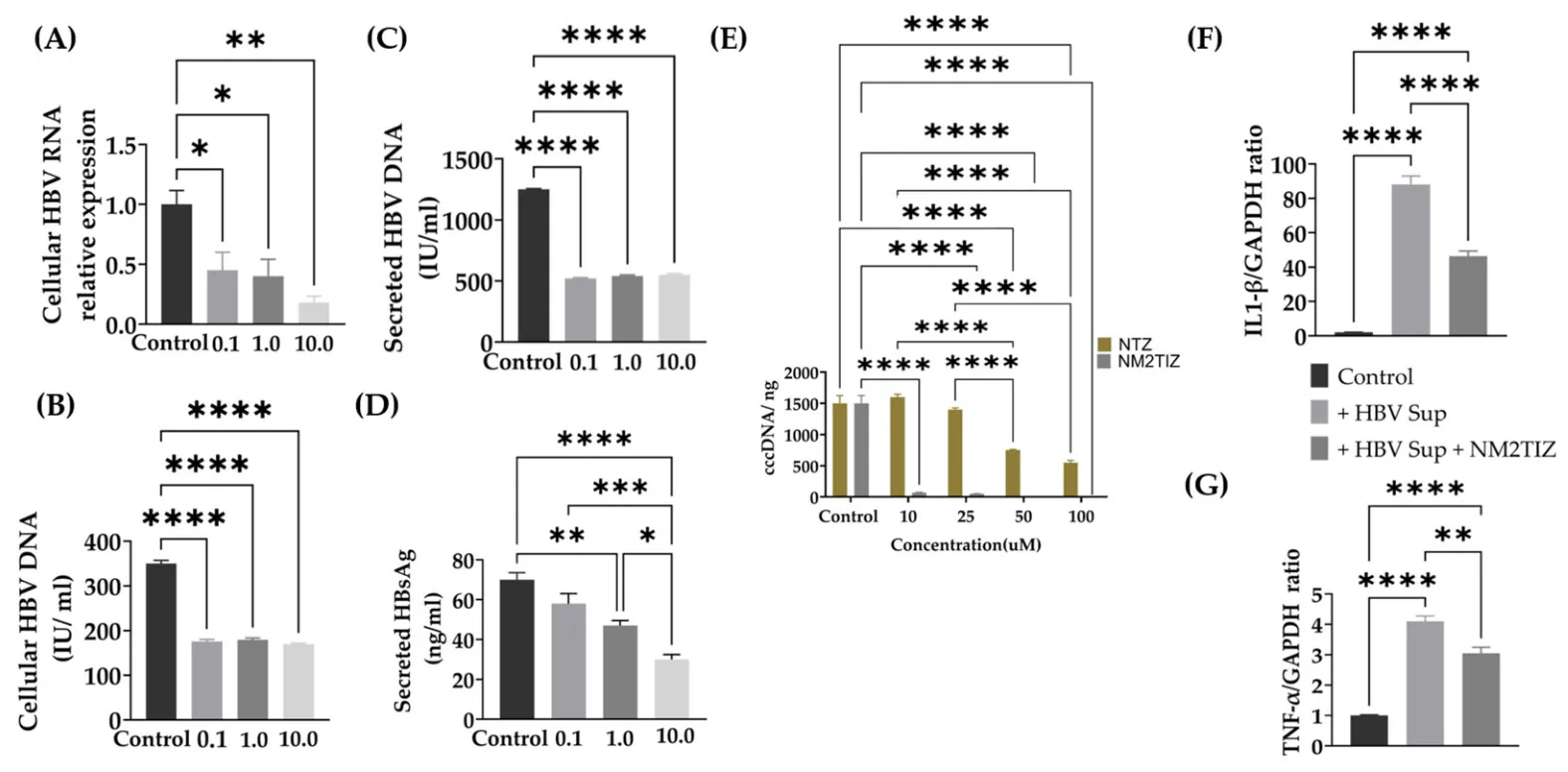
Antiviral and anti-inflammatory effects of NM2TIZ in primary human hepatocytes and macrophages; (**A**–**E**) Antiviral effects measured in HBV-infected PHHs 48 h after drug treatment. Cells were infected with HBV (10^7^ genome equivalents) for 24 h, and non-modified NTZ or NM2TIZ was added at varying concentrations (0.1–10 µM). Cells and supernatants were harvested 48 h later to quantify HBV DNA and HBsAg. (**F**,**G**) Anti-inflammatory activity of NM2TIZ in HBV-stimulated macrophages. MDMs were cultured and exposed to HBV-containing supernatants in the presence or absence of NM2TIZ (10 µM) for 4 h. Cells were collected, and the expression levels of IL-1β and TNF-α mRNA were evaluated. Only significant data are indicated by asterisks (* 0.05 ≥ *p* > 0.01; ** 0.01 ≥ *p* > 0.001; *** 0.001 ≥ *p* > 0.0001; **** *p* ≤ 0.0001).

To assess the anti-HBV activity of NM2TIZ in HBV-infected PHHs, the cells were treated with varying concentrations (0.1, 1.0, and 10 µM) of the prodrug, and HBV RNA, DNA, and HBsAg levels were quantified using RT-PCR and ELISA, respectively. After 8 h of incubation, NM2TIZ significantly suppressed intracellular HBV RNA and HBV DNA levels at all tested concentrations (>50%). Similarly, a marked reduction in secreted HBV DNA, independent of prodrug dose, was observed, whereas secreted HBsAg was significantly suppressed only with 10 µM (Figure 2A–D). NM2TIZ exhibited superior antiviral activity compared to its parent drug NTZ, suggesting improved intracellular drug delivery and efficient activation of the lipophilic prodrug (Figure 2E). To further investigate whether innate immune stimulation was responsible for the observed antiviral effects, human monocyte-derived macrophages (MDMs) were exposed to HBV-containing cell supernatants in the presence or absence of NM2TIZ, after which the relative gene expression of proinflammatory cytokines was measured. NM2TIZ treatment suppressed IL-1β by 1.9-fold as well as TNF-α expression in HBV-stimulated MDMs (Figure 2F,G).

### 3.3. HBV DNA Levels Were Measured After NM2TIZ and NM5TFV Co-Administration

**Figure 3:**
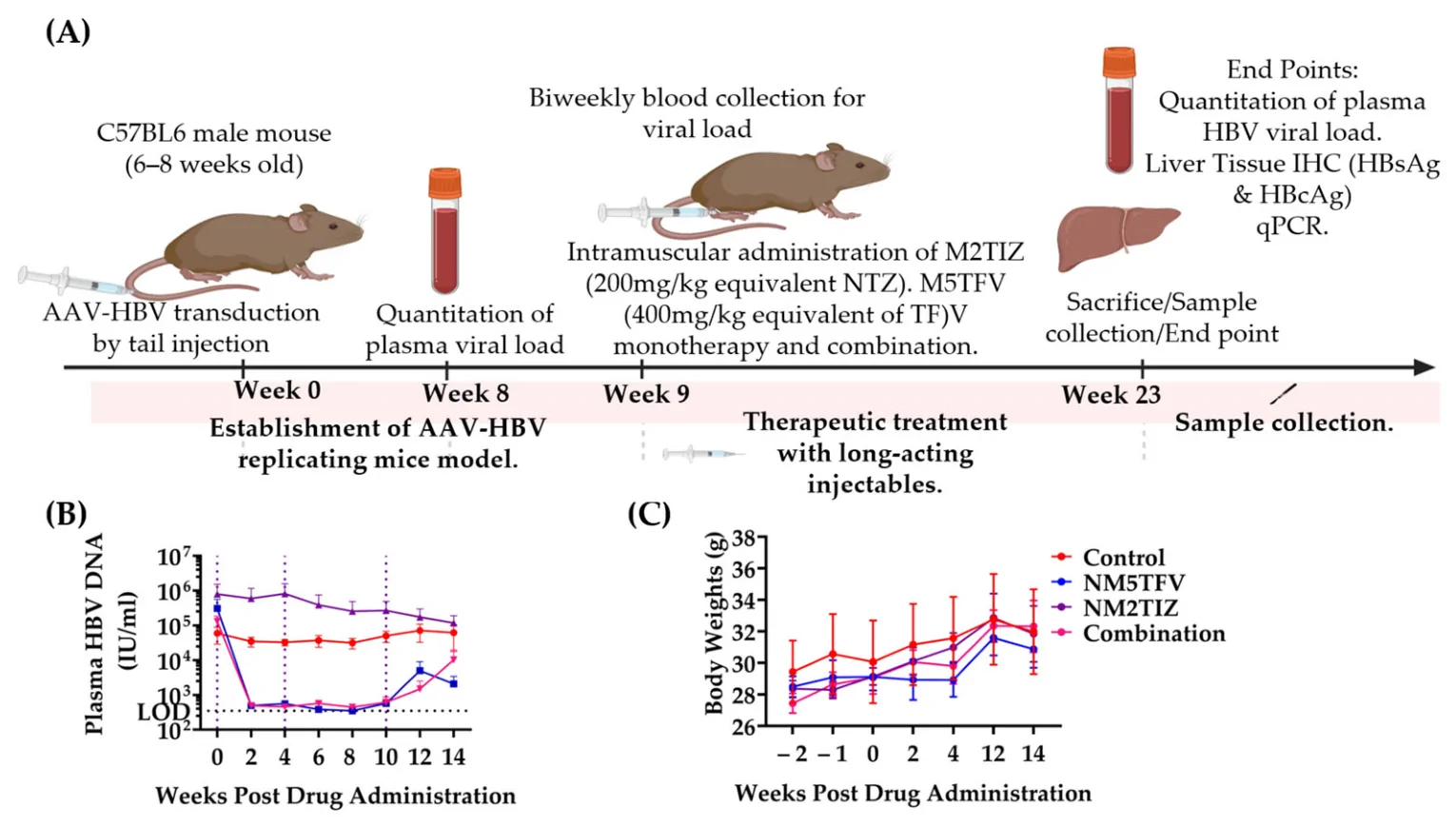
Dual NM5TFV and NM2TIZ and monotherapies in AAV-HBV mice. AAV-HBV mice were administered a single intramuscular dose of NM5TFV (400 mg/kg TFV equivalent), three doses of NM2TIZ (200 mg/kg NTZ equivalent/dose), or a combination of both. The control animals were not treated. (**A**) Experimental layout showing the establishment of HBV replication in immunocompetent mice, administration of NM2TIZ and NM5TFV treatments, and the endpoints. (**B**) NM5TFV monotherapy and combination therapy suppressed HBV replication for eight weeks. (**C**) The NM5TFV monotherapy group showed slight weight loss 2 weeks post-drug administration, which improved at the end of the study, but no significant weight loss was observed in the combination therapy group. The results are shown as the mean ± SEM. One-way ANOVA with Tukey’s multiple-comparison test and Student’s *t*-test were used to analyze the differences in the means between groups, as shown in Table 1.

To evaluate whether NM2TIZ has complementary therapeutic effects when administered in combination with NM5TFV, the prodrugs were administered to immunocompetent mice replicating HBV. For this experiment, AAV-HBV-transduced C57Bl6 mice were treated with NM5TFV monotherapy, NM2TIZ monotherapy, or a combination of NM2TIZ and NM5TFV. The prodrugs were administered as follows: a single intramuscular injection of NM5TFV (400 mg/kg TFV equivalent) and two- and three-monthly doses of NM2TIZ (200 mg/kg TIZ equivalent) in the NM2TIZ alone and combination groups, respectively (Figure 3A). Plasma HBV viral load was measured biweekly for 14 weeks. As anticipated, a single dose of NM5TFV sustained HBV DNA suppression (>2 log) for more than 12 weeks in both the monotherapy and combination therapy groups, but no such effect was observed in the NM2TIZ monotherapy group. A comparison of the absolute log-transformed HBV DNA levels between the NM5TFV monotherapy and combination groups revealed a significant difference only at week 6 (Figure 3B and Table 1). However, an analysis of changes in HBV DNA levels relative to the baseline showed no significant differences between the treatment groups at any time point, indicating comparable overall antiviral suppression. The treatment was well tolerated, and there were no significant differences or changes in the animal body weights (Figure 3C).

### 3.4. NM5TFV and NM2TIZ Combination Therapy Reduces HBV DNA and HBsAg Levels

**Figure 4:**
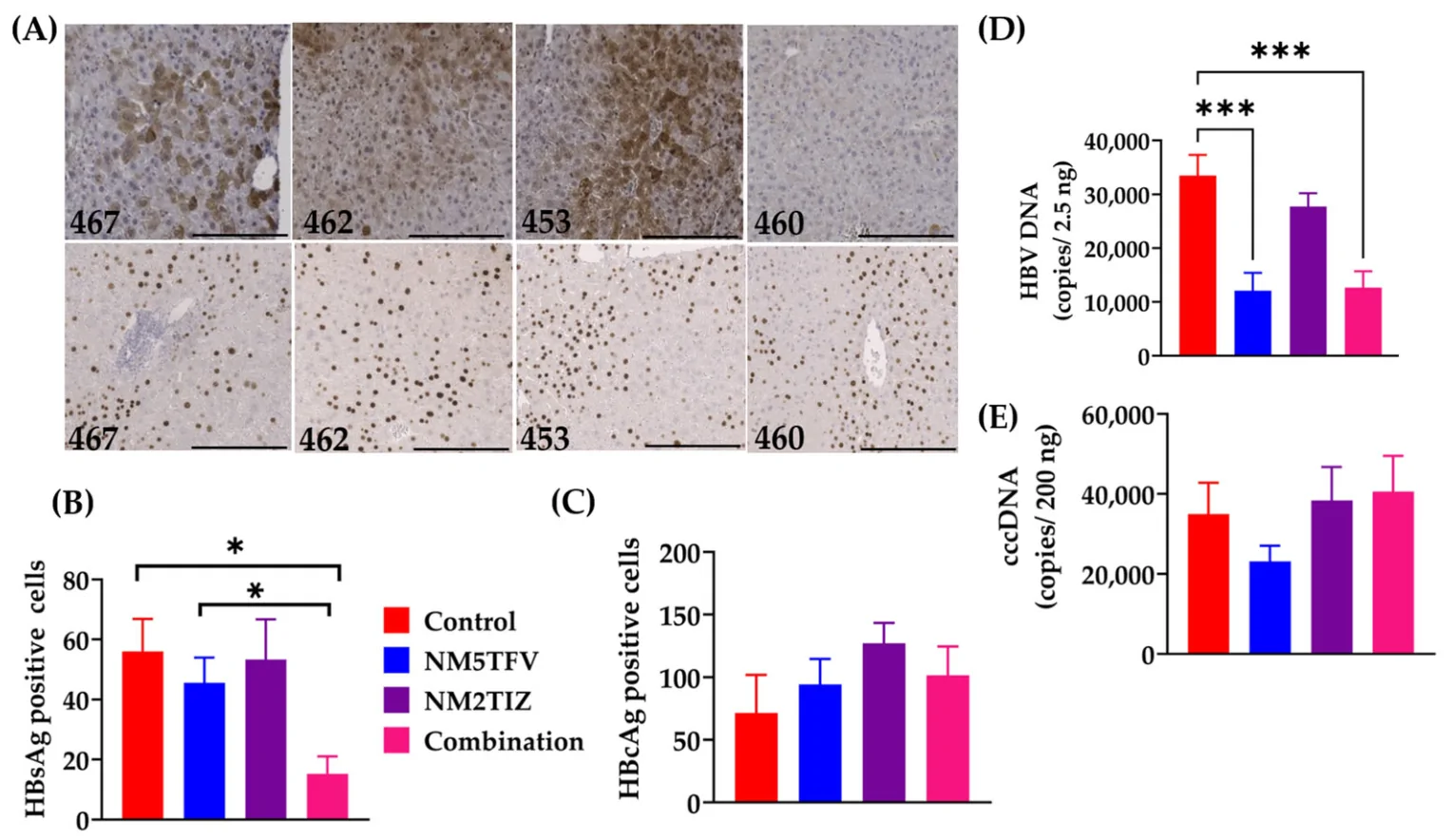
NM2TIZ selectively suppressed HBsAg expression and HBV DNA without affecting cccDNA in the liver tissues of NM5TFV-treated AAV-HBV mice. AAV-HBV-transduced mice were administered monotherapy or combinatorial therapy with a single dose of NM5TFV (400 mg/kg TFV equivalent) and three doses of NM2TIZ (200 mg/kg NTZ-equivalent). The animals were euthanized 14 weeks later. The liver tissue samples were fixed and embedded in paraffin, and 5 μm serial sections were stained for the HBV replication markers HBsAg and HBcAg. Positive signals were visualized using 3′3-diaminobensidine (DAB) (brown). The sections were counterstained with hematoxylin (H). (**A**) Representative montages of HBsAg and HBcAg immunohistochemistry (IHC) staining of liver sections from the control, monotherapy, and combination therapy treatment groups. (**B**,**C**) Mice administered combination therapy significantly suppressed HBsAg-positive cells compared to those administered NM5TFV and NM2TIZ monotherapy and the untreated groups, but no significant difference was observed in HBcAg expression among all groups. (**D**,**E**) NM2TIZ co-administered with NM5TFV showed no additive effect on cccDNA levels in AAV-HBV mouse liver tissues. All images were captured at an original magnification of ×200 (scale bars, 200 µm). The results are expressed as the mean ± SEM for four replicates and analyzed by one-way ANOVA with multiple unpaired *t*-tests. * *p* < 0.05, *** *p* < 0.0005. Only significant data are indicated by asterisks (*).

To evaluate whether combination therapy had synergistic effects on the intrahepatic virologic burden, hepatic HBV DNA and cccDNA were quantified using qPCR (Figure 4D,E). Our findings showed that total HBV DNA levels were significantly reduced by NM5TFV monotherapy and in combination groups, but no significant effect was observed between the control and NM2TIZ monotherapy groups. In addition, cccDNA levels were comparable between the control and treatment groups, suggesting that combination therapy had no direct effect on intrahepatic viral replication or cccDNA persistence in this animal model. Further immunohistochemistry studies were conducted to probe alterations in the intrahepatic expression of viral antigens, HBsAg, and HBcAg (Figure 4A–C). Surprisingly, while HBcAg expression levels were similar in the control and treatment groups, we observed a marked reduction in HBsAg-positive staining in the combination therapy group compared with the control and monotherapy groups. This indicates that combination therapy selectively decreases intrahepatic HBsAg accumulation through a distinct pathway independent of the pgRNA-HBV DNA reverse transcription mechanism.

### 3.5. Complementary Immune Pathways Mitigating HBV-Induced Inflammation and Fibrosis

**Figure 5:**
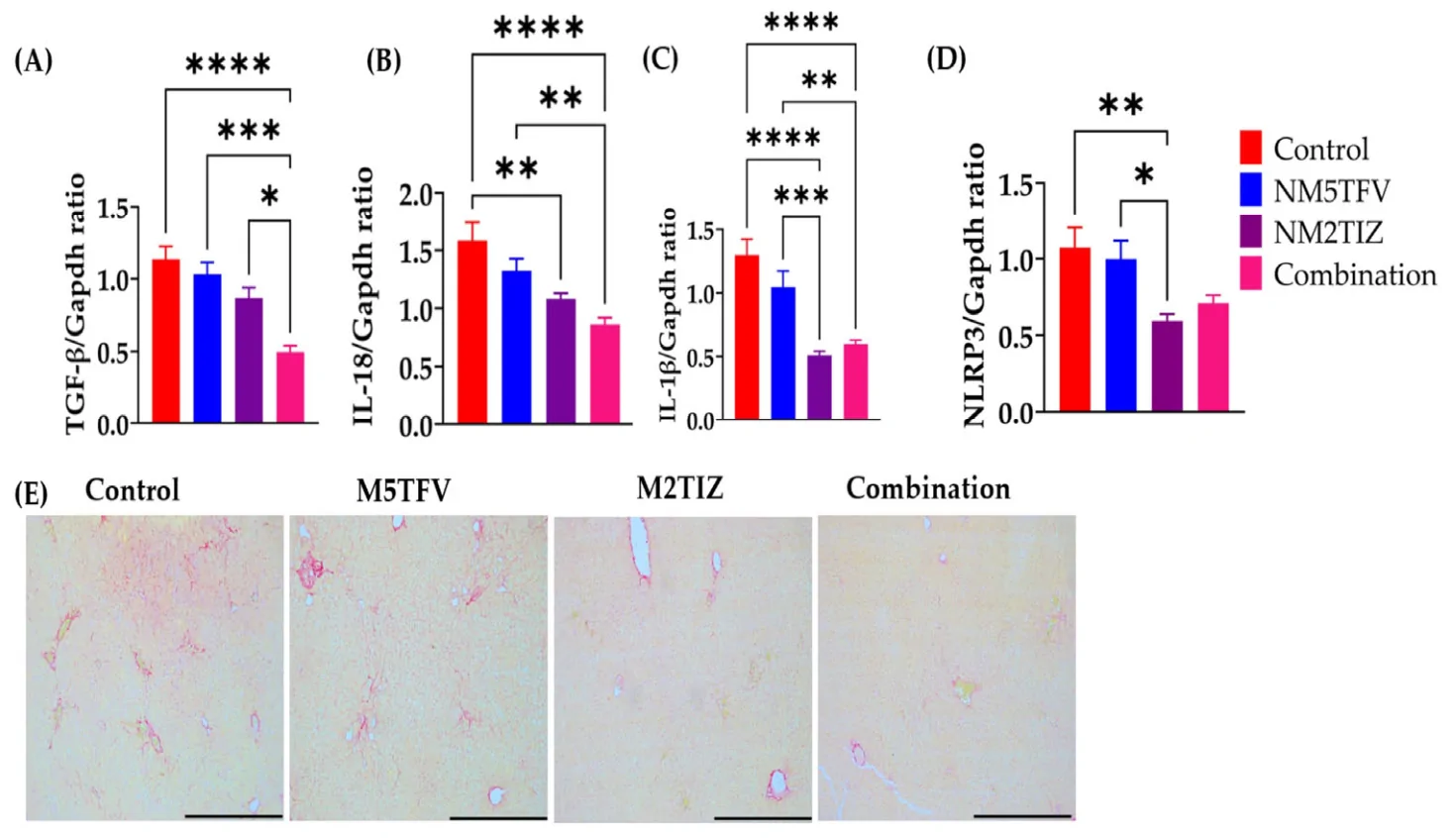
NM2TIZ anti-inflammatory and anti-fibrotic phenotypes in the liver tissues of NM5TFV-treated AAV-HBV mice. RNA samples were purified from frozen liver tissues of AAV-HBV-transduced mice administered NM5TFV (400 mg/kg TFV equivalent), NM2TIZ (200 mg/kg NTZ equivalent), the NM5TFV + NNM2TIZ combination, and control untreated mice. The gene expression levels of profibrotic and inflammasome-related markers, TGF-β, IL-18, IL-1β, and NLRP3, were determined by RT-PCR. (**A**) NM2TIZ and NM5TFV combination therapy significantly suppressed the gene expression of the pro-fibrotic marker TGF-β, compared with monotherapy and control groups. (**B**) NM2TIZ, NM5TFV monotherapy, and the combination had no significant effect on IL-18, and the expression level was comparable to that of the control. (**C**) IL-1β gene expression was significantly downregulated in the NM2TIZ monotherapy and combination therapy groups compared with the other groups. (**D**) NLRP3 expression was significantly lower in the NM2TIZ monotherapy group than in the other groups. (**E**) Collagen deposition was evaluated by Sirius Red staining of frozen liver sections from the experimental mice. All images were captured at an original magnification of ×100 (scale bars, 100 µm). Data are represented as mean ± SEM, *n* = 5. Only significant data are indicated by asterisks (* 0.05 ≥ *p* > 0.01; ** 0.01 ≥ *p* > 0.001; *** 0.001 ≥ *p* > 0.0001; **** *p* ≤ 0.0001).

TIZ has been shown to inhibit profibrogenic and proinflammatory cytokines in previous studies [25,26] and in the current in vitro data (Figure 2F,G). Therefore, we investigated whether the NM2TIZ prodrug retained its immune stimulatory properties following chemical modification and whether its activity afforded compensatory attenuation of HBV-associated inflammation during tenofovir therapy. To achieve this, the gene expression profiles of profibrotic markers, TGF-β, inflammasome-mediated cytokines (IL-1β and IL-18), and an inflammasome receptor, NLRP3, in liver tissues were quantified using qPCR in the control and treatment groups. Compared to the control and monotherapy groups, NM2TIZ and NM5TFV combination therapy exhibited a synergistic effect in downregulating TGF-β expression (Figure 5A). Unlike NM5TFV monotherapy, which showed no anti-inflammatory effect, NM2TIZ significantly suppressed IL-18 and IL-1β levels in the monotherapy and combination groups (Figure 5B,C). However, NLRP3 was only suppressed in both NM2TIZ monotherapy groups, but not in the NM5TFV monotherapy or combination groups (Figure 5D). To further investigate whether the observed anti-inflammatory effect contributes to fibrogenesis, we assessed collagen deposition in liver sections using Sirius Red staining (Figure 5E). Consistent with the anti-inflammatory findings, histological examination revealed extensive collagen deposition in HBV-infected control and NM5TFV monotherapy mice, whereas combination-treated animals exhibited markedly reduced collagen accumulation and preservation of hepatic architecture. Collectively, NM2TIZ affords compensatory remediation of inflammation and fibrotic progression when combined with NM5TFV.

## 4. Discussion

Recent advancements in therapeutic strategies for combating cirrhosis and hepatocellular carcinoma in CHB have shifted from frequently dosed monotherapies to leveraging LA approaches for delivering multi-mechanistic compounds capable of achieving a functional cure. Notably, the sustained antiviral efficacy of NM5TFV observed in the AAV-HBV mouse model used in this study is consistent with our previously published findings in HBV-transgenic and liver-humanized mice [7,8], further confirming its robust potential in infrequent dosing therapies. Hence, months-long LA approaches for nucleos(t)ide analog administration are desirable for ease and improved participation in clinical investigations.

The novelty of NM2TIZ lies in its bioavailability compared with that of TIZ and NTZ. This improvement was achieved through the esterification of TIZ with stearoyl chloride, imparting increased lipophilicity required for enhanced intracellular drug delivery. Although NM2TIZ was administered monthly and does not offer synchronized dosing intervals with the every-three-month NM5TFV formulation, the co-administration of both prodrugs afforded greater therapeutic benefits than the standalone monotherapy approach. Future studies should explore the pharmacokinetics of LA TIZ derivatives and synchronized doses to further simplify treatment.

Since a functional cure for CHB is defined as undetectable HBV DNA levels and HBsAg loss [27,28], we evaluated the effects of NM2TIZ on these markers in PHHs and AAV-HBV-transduced mice. Consistent with previous reports, NM2TIZ alone robustly suppressed HBV DNA, cccDNA, and HBsAg levels in PHHs, further supporting the concept that hepatocytes are equipped with an intrinsic immune repertoire capable of mounting strong antiviral effects upon stimulation with NM2TIZ [29,30]. In contrast to the encouraging in vitro findings, NM2TIZ monotherapy had no significant effect on hepatic HBV DNA, HBcAg, cccDNA, or HBsAg levels in the AAV-HBV mouse model. This unexpected result could be attributed to species-specific differences in antiviral repertoire or distinct disease conditions modeled by each experimental system. Unlike the simplified PHH culture system, which closely mimics acute natural infection, the AAV-HBV mouse lacks human-specific factors required for cccDNA maintenance, and high viremia was already established before therapy intervention. AAV-HBV supports long-term viral persistence, and continuous antigen production (HBsAg and HBeAg) drives immune dysfunction and CHB-associated abnormalities, including dampened innate immune responsiveness, CD8^+^ T cell exhaustion, a switch to a tolerogenic immune profile, and fibrogenesis [31,32,33,34]. Although it has previously been reported that ccc_Ro8 effectively suppressed cccDNA and HBV DNA in HBV circle mice, the result was not reproduced in naturally infected humanized mice at the same levels [35]. The complex immune dysregulated crosstalk and fibrotic remodeling in the AAV-HBV mouse model could be responsible for the limited antiviral activity of NM2TIZ compared with observations from prior studies [31,34]. In addition, suboptimal drug delivery could also contribute to the ablated NM2TIZ efficacy observed in our CHB mouse model. One of the limitations of this study is the lack of quantitation of TIZ levels in plasma and tissues following drug administration. Future studies will characterize the pharmacokinetic profile of NM2TIZ in rat models with sufficient muscle mass for remodeling drug absorption and release following intramuscular administration.

Notably, significant HBsAg loss occurred when NM2TIZ was co-administered with NM5TFV, demonstrating a distinct yet complementary interaction between the two prodrugs. Compared with the twice-daily NTZ regimen used in previous clinical studies, NM2TIZ achieved a sustained reduction in HBsAg activity with substantially less frequent dosing over a relatively short 14-week treatment period [13]. Notably, the presence of a highly potent NM5TFV in the combination group appears to significantly suppress HBV DNA synthesis, with no potentiation by NM2TIZ. However, unlike NM5TFV, NM2TIZ demonstrated a potent anti-inflammatory property potentiated by breaking immune tolerance due to removal of HBsAg. Surprisingly, the combination group showed preferential suppression of HBsAg without affecting other HBV replication markers, including HBcAg, HBV DNA, and cccDNA, suggesting a distinct inhibitory pathway independent of conventional cccDNA destabilization. Although the selective suppression of HBsAg in the combination group warrants further investigation, we speculate that NM2TIZ minimizes the stress in the endoplasmic reticulum (ER), previously linked to the prevention of HBsAg maturation [18,36]. Additionally, although downstream stimulation of the adaptive arm of the immune system was not investigated in this preliminary study, the reduced viral load could have reduced antigen-mediated immune suppression, thereby restoring immune signaling and production of anti-HBsAg antibodies needed for HBsAg clearance. Given the disparity in pathogen receptor sensitivity between humans and mice [37], the additive antiviral activity of NM2TIZ observed in this study may be underestimated, warranting future mechanistic studies in humanized models to improve clinical translatability.

Considering the contributions of both viral and host pathways in HBV pathogenesis, progression, and associated immune pathologies [38], dual-acting therapeutic strategies are desirable to overcome the evasive defense mechanisms of the stealth virus. Several studies have demonstrated that viral targeting therapies, such as entry inhibitors, nucleos(t)ide analogs, gene therapies, or capsid inhibitors, attenuate suppression of viral nucleic acids and proteins. However, standalone therapies rarely remediate the immune dysfunction and collateral liver damage caused by already established viremia [5,39]. Notably, beyond prolonging the duration of the parent compound’s action and conferring antiviral activity, NM2TIZ uniquely ameliorates hepatic inflammation and liver injury in AAV-HBV mice, complementing the LA antiviral activity of the previously published NM5TFV. From our findings, the gene expression profiles of IL-1β and IL-18, major inflammasome-related cytokines, were markedly downregulated in the NM2TIZ and NM5TFV combination group, while NM5TFV showed no such anti-inflammatory activity. Although the decline in the NLRP3 inflammasome receptor was only numerically suppressed in the combination group, we observed its significant downregulation in the NM2TIZ but not NM5TFV monotherapy group. Interestingly, simultaneous NM2TIZ and NM5TFV treatment substantially preserved liver tissue integrity, reflected by attenuated *tgfb* gene expression and diminished collagen deposition compared with NM5TFV alone. Compared to existing NA monotherapy, the NM2TIZ and NM5TFV combination strategies could potentially improve treatment outcomes and pathological bottlenecks in the HBV disease continuum, providing a balanced treatment approach. Alternative combinatorial approaches, namely, the integration of next-generation HBV therapeutics, such as bepirovirsen, in NM5TFV therapy with or without NM2TIZ, could enhance antiviral responses and improve therapeutic outcomes [40,41].

## 5. Conclusions

Combinations of LA NM2TIZ and NM5TFV prodrug formulations promote antiviral, anti-inflammatory, anti-fibrotic, and innate immune restorative benefits in AAV-HBV mice with complementary mechanisms of action. This strategy, by combining LA anti-HBV interventions with an anti-inflammatory/anti-fibrotic component, may simplify treatment regimens to improve clinical outcomes.

## 6. Patents

B.J.E. is a named inventor on a pending patent application that covers ultra-LA phosphonate prodrugs of nucleos(t)ide analogs (US Provisional Patent Application No. 63/540,486, Application Serial No.: PCT/US2024/048259).

## Figures and Tables

**Table 1:** NM5TFV and NM2TIZ therapies in AAV-HBV mice.

^1^ Weeks 1.	^2^ HBV DNA log_10_				^3^ Viral Load Reduction			^4^ Significance of Drug Effects, *p* Values			
	Control	NM5TFV	NM2TIZ	^5^ TFV + TIZ	NM5TFV	NM2TIZ	TFV + TIZ	NM5TFV	NM2TIZ	TFV + TIZ	NM5TFV vs. Combination
0	4.66	4.71	4.89	4.83							
2	4.50	2.69	4.72	2.66	−2.02	−0.17	−2.16	0.0029	0.7789	0.0002	0.7158
4	4.50	2.72	5.04	2.64	−1.99	0.15	−2.19	0.0033	0.7799	0.0001	0.4079
6	4.54	2.58	4.73	2.74	−2.13	−0.15	−2.08	0.0021	0.7784	0.0002	0.0373
8	4.46	2.54	4.65	2.62	−2.17	−0.24	−2.21	0.0019	0.6504	0.0001	0.3466
10	4.65	2.69	4.86	2.68	−2.02	−0.03	−2.15	0.0033	0.9597	0.0002	0.9658
12	4.73	3.09	4.77	2.86	−1.62	−0.11	−1.97	0.0259	0.8271	0.0010	0.6099
14	4.55	2.99	4.78	3.14	−1.72	−0.11	−1.68	0.0128	0.8263	0.0107	0.7488

^1.^ Week after drug administration. ^2^ Mean values are presented. ^3^ Viral load adjusted to baseline at week 0. ^4^ *p* values of NM2TIZ-treated, NM5TFV monotherapy, and combination-treated mice; two-way ANOVA, Tukey’s multiple-comparison test, and Student’s *t*-test. ^5^ Abbreviated NM5TFV and NM2TIZ formulations.

## Data Availability

All non-restricted data are published in the manuscript.

## Abbreviations

The following abbreviations are used in this manuscript:

NTZ	Nitazoxanide
cccDNA	Covalently closed circular DNA
M2TIZ	Tizoxanide prodrug
M5TFV	Tenofovir prodrug
NM2TIZ	Nanoformulated tizoxanide prodrug
NM5TFV	Nanoformulated tenofovir prodrug

## References

[1] World Health Organization (2025). Hepatitis B. https://www.who.int/en/news-room/fact-sheets/detail/hepatitis-b.

[2] Kowdley K.V., Wang C.C., Welch S., Roberts H., Brosgart C.L. (2012). Prevalence of chronic hepatitis B among foreign-born persons living in the United States by country of origin. Hepatology.

[3] Platt L., French C.E., McGowan C.R., Sabin K., Gower E., Trickey A., McDonald B., Ong J., Stone J., Easterbrook P. (2020). Prevalence and burden of HBV co-infection among people living with HIV: A global systematic review and meta-analysis. J. Viral Hepat..

[4] Terrault N.A., Bzowej N.H., Chang K.M., Hwang J.P., Jonas M.M., Murad M.H. (2016). AASLD guidelines for treatment of chronic hepatitis B. Hepatology.

[5] Lim S.G., Baumert T.F., Boni C., Gane E., Levrero M., Lok A.S., Maini M.K., Terrault N.A., Zoulim F. (2023). The scientific basis of combination therapy for chronic hepatitis B functional cure. Nat. Rev. Gastroenterol. Hepatol..

[6] Terrault N.A., Lok A.S.F., McMahon B.J., Chang K.-M., Hwang J.P., Jonas M.M., Brown R.S., Bzowej N.H., Wong J.B. (2018). Update on prevention, diagnosis, and treatment of chronic hepatitis B: AASLD 2018 hepatitis B guidance. Hepatology.

[7] Raut S.S., Das S., Bybee G., Chava H., Ogunnaike M., Wang W., Pathania A.S., Hanson B.W., Le N.T.H., Cohen S.M. (2025). A scalable ultra-long-acting tenofovir phosphonate prodrug sustains HBV suppression. Sci. Adv..

[8] Das S., Wang W., Ganesan M., Fonseca-Lanza F., Cobb D.A., Bybee G., Sun Y., Guo L., Hanson B., Cohen S.M. (2022). An ultralong-acting tenofovir ProTide nanoformulation achieves monthslong HBV suppression. Sci. Adv..

[9] Allweiss L., Dandri M. (2017). The Role of cccDNA in HBV Maintenance. Viruses.

[10] Hsu Y.C., Huang D.Q., Nguyen M.H. (2023). Global burden of hepatitis B virus: Current status, missed opportunities and a call for action. Nat. Rev. Gastroenterol. Hepatol..

[11] Zoulim F., Durantel D. (2015). Antiviral therapies and prospects for a cure of chronic hepatitis B. Cold Spring Harb. Perspect. Med..

[12] Singh N., Narayan S. (2011). Nitazoxanide: A Broad Spectrum Antimicrobial. Med. J. Armed Forces India.

[13] Rossignol J.F. (2016). Nitazoxanide, a new drug candidate for the treatment of Middle East respiratory syndrome coronavirus. J. Infect. Public Health.

[14] Korba B.E., Montero A.B., Farrar K., Gaye K., Mukerjee S., Ayers M.S., Rossignol J.-F. (2008). Nitazoxanide, tizoxanide and other thiazolides are potent inhibitors of hepatitis B virus and hepatitis C virus replication. Antivir. Res..

[15] Sekiba K., Otsuka M., Ohno M., Yamagami M., Kishikawa T., Suzuki T., Ishibashi R., Seimiya T., Tanaka E., Koike K. (2019). Inhibition of HBV Transcription From cccDNA With Nitazoxanide by Targeting the HBx-DDB1 Interaction. Cell Mol. Gastroenterol. Hepatol..

[16] Korba B.E., Elazar M., Lui P., Rossignol J.F., Glenn J.S. (2008). Potential for hepatitis C virus resistance to nitazoxanide or tizoxanide. Antimicrob. Agents Chemother..

[17] Keeffe E.B., Rossignol J.F. (2009). Treatment of chronic viral hepatitis with nitazoxanide and second generation thiazolides. World J. Gastroenterol..

[18] Piacentini S., Riccio A., Santopolo S., Pauciullo S., La Frazia S., Rossi A., Rossignol J.-F., Santoro M.G. (2023). The FDA-approved drug nitazoxanide is a potent inhibitor of human seasonal coronaviruses acting at postentry level: Effect on the viral spike glycoprotein. Front. Microbiol..

[19] Sekiba K., Otsuka M., Ohno M., Kishikawa T., Yamagami M., Suzuki T., Ishibashi R., Seimiya T., Tanaka E., Koike K. (2018). DHX9 regulates production of hepatitis B virus-derived circular RNA and viral protein levels. Oncotarget.

[20] Tan Y.C., Shen L., Khine H.H.T.W., Tay A., Andrenada C., Rossignol J.F., Rossignol C., Lim S.G. Randomized double-blind study of Nitazoxanide for virologically suppressed HBeAg negative Chronic Hepatitis B. Proceedings of the International Liver Congress™ 2022 (European Association for the Study of the Liver).

[21] Sood S., Maddiboyina B., Rawat P., Garg A.K., Foudah A.I., Alam A., Aldawsari H.M., Riadi Y., Singh S., Kesharwani P. (2021). Enhancing the solubility of nitazoxanide with solid dispersions technique: Formulation, evaluation, and cytotoxicity study. J. Biomater. Sci. Polym. Ed..

[22] Du Y., Broering R., Li X., Zhang X., Liu J., Yang D., Lu M. (2021). In Vivo Mouse Models for Hepatitis B Virus Infection and Their Application. Front. Immunol..

[23] Tao M.H., Huang Y.H., Chou H.Y., Fang C.C. (2011). A mouse model of T cell dysfunction in chronic Hepatitis B. J. Immunol..

[24] Ganesan M., Wang W., Mathews S., Makarov E., New-Aaron M., Dagur R.S., Malo A., Protzer U., Kharbanda K.K., Casey C.A. (2022). Ethanol attenuates presentation of cytotoxic T-lymphocyte epitopes on hepatocytes of HBV-infected humanized mice. Alcohol Clin. Exp. Res..

[25] Shou J., Kong X., Wang X., Tang Y., Wang C., Wang M., Zhang L., Liu Y., Fei C., Xue F. (2019). Tizoxanide Inhibits Inflammation in LPS-Activated RAW264.7 Macrophages via the Suppression of NF-κB and MAPK Activation. Inflammation.

[26] Liu K.X., Wang Z.Y., Ying Y.T., Wei R.M., Dong D.L., Sun Z.J. (2024). The antiprotozoal drug nitazoxanide improves experimental liver fibrosis in mice. Biochem. Pharmacol..

[27] Wong G.L.H., Gane E., Lok A.S.F. (2022). How to achieve functional cure of HBV: Stopping NUCs, adding interferon or new drug development?. J. Hepatol..

[28] Lok A.S., Zoulim F., Dusheiko G., Ghany M.G. (2017). Hepatitis B cure: From discovery to regulatory approval. J. Hepatol..

[29] Woo Y., Ma M., Okawa M., Saito T. (2024). Hepatocyte Intrinsic Innate Antiviral Immunity against Hepatitis Delta Virus Infection: The Voices of Bona Fide Human Hepatocytes. Viruses.

[30] Zhou Z., Xu M.J., Gao B. (2016). Hepatocytes: A key cell type for innate immunity. Cell Mol. Immunol..

[31] Yang D., Liu L., Zhu D., Peng H., Su L., Fu Y.-X., Zhang L. (2014). A mouse model for HBV immunotolerance and immunotherapy. Cell Mol. Immunol..

[32] Conceição-Neto N., Pierson W., Han Q., Yao Z., Wu Q., Dockx K., Aerts L., De Maeyer D., Beyens M., Berge K.V.D. (2025). AAV-HBV mouse model replicates the intrahepatic immune landscape of chronic HBV patients at single-cell level. Front. Immunol..

[33] Wang L., Zeng X., Wang Z., Fang L., Liu J. (2023). Recent advances in understanding T cell activation and exhaustion during HBV infection. Virol. Sin..

[34] Seki E., Schwabe R.F. (2015). Hepatic inflammation and fibrosis: Functional links and key pathways. Hepatology.

[35] Wang L., Zhu Q., Zhang J.D., Zhang Y., Ni X., Xiang K., Jiang J., Li B., Yu Y., Hu H. (2023). Discovery of a first-in-class orally available HBV cccDNA inhibitor. J. Hepatol..

[36] Piacentini S., La Frazia S., Riccio A., Pedersen J.Z., Topai A., Nicolotti O., Rossignol J.-F., Santoro M.G. (2018). Nitazoxanide inhibits paramyxovirus replication by targeting the Fusion protein folding: Role of glycoprotein-specific thiol oxidoreductase ERp57. Sci. Rep..

[37] Ariffin J.K., Sweet M.J. (2013). Differences in the repertoire, regulation and function of Toll-like Receptors and inflammasome-forming Nod-like Receptors between human and mouse. Curr. Opin. Microbiol..

[38] Lebossé F., Testoni B., Fresquet J., Facchetti F., Galmozzi E., Fournier M., Hervieu V., Berthillon P., Berby F., Bordes I. (2017). Intrahepatic innate immune response pathways are downregulated in untreated chronic hepatitis B. J. Hepatol..

[39] Bertoletti A., Ambike S.S., Le Bert N., Binayke A.A. (2026). Therapeutic vaccination for chronic hepatitis B: Why most vaccines failed and what may work. Antivir. Res..

[40] Hou J., Lim S.G., Buti M., Yuen M.-F., Gane E., Lampertico P., Terrault N., Nguyen H., Yim H.J., Xie Q. (2026). Phase 3 Results of Bepirovirsen Treatment for Chronic Hepatitis B Virus Infection. N. Engl. J. Med..

[41] Hui R.W., Mak L.Y., Fung J., Seto W.K., Yuen M.F. (2025). Prospect of emerging treatments for hepatitis B virus functional cure. Clin. Mol. Hepatol..

